# Constitutive Analysis of the Deformation Behavior of Al-Mg-Si Alloy Under Various Forming Conditions Using Several Modeling Approaches

**DOI:** 10.3390/ma18051121

**Published:** 2025-03-01

**Authors:** Bandar Alzahrani, Ali Abd El-Aty, Yong Xu, Yong Hou, Shi-Hong Zhang, Alamry Ali, Mohamed M. Z. Ahmed, Abdallah Shokry

**Affiliations:** 1 Department of Mechanical Engineering, College of Engineering at Al Kharj, Prince Sattam Bin Abdulaziz University, Al Kharj 11942, Saudi Arabia; 2 Institute of Metal Research, Chinese Academy of Sciences, Shenyang 110016, China; 3 Shenyang Key Laboratory for Aerospace Complex Components Precision Manufacturing, Shenyang 110016, China; 4 Institute of Forming Technology and Lightweight Components (IUL), TU Dortmund University, 44227 Dortmund, Germany; 5 Department of Mechanical Engineering, Faculty of Engineering, Fayoum University, Fayoum 63514, Egypt

**Keywords:** deformation behavior, elevated temperatures, strain rate, physical models, Crystal Plasticity modeling, phenomenological models

## Abstract

The hot-flow behaviors of Al-Mg-Si alloy are complex because they depend on ε, $\dot{\varepsilon }$, and T. Hence, it is vital to understand and determine the Al-Mg-Si alloy’s flow behaviors under several deformation conditions. Therefore, in this study, Crystal Plasticity (CP) modeling, modified Zerilli–Armstrong (MZA), and two JC models were developed to precisely determine the hot deformation behaviors of this alloy. The reliability and predictability of these models were evaluated via comparisons of the determined and experimental results acquired in the $\dot{\varepsilon }$ range of 10^−3^ to 1 s^−1^ and T range of 400–550 °C. Additionally, statistical parameters including the RMSE, AARE, and R were utilized to assess these models’ reliability for determining this alloy’s flow behaviors under several forming conditions. By analyzing these statistical parameters and comparing the predicted and experimental stresses, it can be concluded that the flow stresses predicted by the CP modeling and S2-MJC model exhibit a strong alignment with the experimental flow stresses. This contrasts with the results from the MZA and S1-MJC models. These results are attributed to the ability of CP modeling to couple the microstructure state of this alloy and the interactions between ε and $\dot{\varepsilon }$ on the one hand and between T, $\dot{\varepsilon }$, and ε on the other hand, facilitated by a comprehensive set of parameters that link the dynamic recovery and softening mechanisms components in the S2‐MJC model.

## 1. Introduction

Al alloys are widely recognized as a green material because of their exceptional environmental benefits and sustainable characteristics, making them highly valued across construction, automotive, and aerospace industries [1]. A key factor in their sustainability is their high recyclability as Al can be recycled indefinitely without losing its properties [2]. Recycling Al requires only 5% of the energy used in primary production processes such as the Hall–Héroult and Bayer methods, significantly reducing greenhouse gas emissions and promoting a circular economy [3].

In addition to recyclability, Al alloys offer a combination of valuable attributes. Their low density reduces the weight in large structures and vehicles, improving efficiency and reducing material consumption [4]. Coupled with high strength and corrosion resistance, Al alloys provide long-term durability and structural integrity, even in harsh environments such as marine settings [5]. These characteristics make Al alloys ideal for applications in high-rise buildings, where they ensure durability and resistance to corrosion; large-span structures, such as stadiums and airports, where their strength-to-weight ratio allows for open, functional designs; bridge construction, where they reduce structural weight and maintenance costs; and shipbuilding, where their corrosion resistance and lightweight nature improve fuel efficiency and minimize maintenance in challenging marine environments [6,7,8].

By combining environmental benefits, energy efficiency, and versatility, Al alloys directly contribute to advancing Sustainable Development Goals (SDGs), including SDG 12, by promoting resource efficiency, reducing material waste, and encouraging circular economy practices. Additionally, their lightweight nature and high strength enable the production of energy-efficient vehicles and sustainable infrastructure, supporting SDG 9 and SDG 11. Al alloys support sustainable practices and drive innovation in modern industries, solidifying their importance in achieving global sustainability goals [9,10,11].

The Al-Mg-Si 6xxx-series alloy has gained significant attention in the automobile and aerospace industries because of its high specific strength, excellent formability, and excellent corrosion resistance, making it ideal for lightweight structural components [12,13,14]. Its use supports improved fuel efficiency, reduced emissions, and sustainable manufacturing practices, aligning with industry priorities [15]; however, its low formability at room temperature poses challenges in shaping complex components, leading to material waste and limiting its broader industrial applications in designs requiring high precision and minimal waste [13,14].

High-speed and elevated-temperature deformation techniques are commonly employed to enhance alloy formability, enabling the production of complex shapes while minimizing defects such as cracks [15,16,17,18,19,20,21,22,23]. For example, Abd El-Aty et al. [16] examined the tensile behavior of several Al alloys at high strain rates. They reported that high-speed forming is an effective technique for enhancing the formability of Al alloys at room temperature. Elevated-temperature forming is often preferred because it significantly improves alloy formability, facilitating deformation without fracturing and reducing the risk of defects [24]. This method allows for better control over the forming process and creates more complex geometries while lowering the stress on tools and equipment, extending their lifespan and reducing production costs [25]. In contrast, while effective for certain applications, high-speed forming can lead to challenges such as non-uniform strain distribution and a greater risk of surface defects due to rapid force application, making it harder to control [26]. For these reasons, elevated-temperature forming is a more reliable and controlled option in many industrial applications.

The flow behavior of Al alloys at elevated temperatures is complex because of interactions among T, $\dot{\varepsilon }$, and microstructural changes during deformation [27,28,29,30]. Therefore, understanding their deformation behaviors is critical for optimizing the forming process to ensure that components retain their mechanical properties and dimensional accuracy. For Al-Mg-Si alloys, experimental investigations under various T, $\dot{\varepsilon }$, and loading conditions are essential for evaluating their mechanical properties and deformation behavior. However, due to the complexity of hot deformation, computational tools are also necessary to predict and enhance material performance in industrial applications [29].

Advanced modeling techniques coupled with the FEM are essential for simulating and predicting the deformation behavior of metallic materials. The FEM enables manufacturers to optimize the strain distribution, temperature effects, and microstructural changes in a virtual environment before practical implementation [28,29,30]. Recent advances have led to the development of various constitutive models to predict the hot-flow behavior of metals, including phenomenological such as the JC model, physically based such as Zerilli–Armstrong (ZA) and CP modeling, as well as artificial neural network (ANN) models [31,32,33,34,35,36,37,38,39,40,41,42,43,44,45,46,47,48,49,50,51,52,53,54,55,56,57,58,59,60,61,62].

From the aforementioned discussion, it is summarized that predicting the hot-flow behaviors of Al-Mg-Si alloys is crucial for analyzing the influence of the interactions T, $\dot{\varepsilon }$, and microstructural changes during the deformation process. This understanding is essential for optimizing the forming processes to ensure that components maintain their mechanical properties and dimensional accuracy under high-temperature conditions. Thus, this study aims to develop and evaluate four advanced constitutive models including CP modeling, the MZA model, and two modified Johnson–Cook (JC) models to predict the hot deformation behavior of Al-Mg-Si alloys accurately. This work provides valuable insights for improving the efficiency and precision of industrial forming processes. The accuracy and predictability of these models are evaluated by comparing their results with the experimental results obtained from the investigation of Ghosh et al. [54]. Several statistical parameters, including R, AARE, and RMSE, were subsequently used to further validate the reliability of the proposed models in predicting the hot-flow behaviors of Al-Mg-Si alloys. A detailed discussion of each constitutive model is presented in the following sections.

## 2. Experimental Section

In the present study, the experimental results obtained by Ghosh et al. [54] were used to verify the predictability and reliability of the developed models. Their investigation explored the hot deformation behavior of an Al-Mg-Si alloy (AA6082) via a Gleeble-3800 thermomechanical simulator under 16 combinations of $\dot{\varepsilon }$ ranging from 0.001/s to 1/s as well as T from 400 to 550 °C. The flow curves revealed that the deformation behavior aligns with typical alloys under similar elevated conditions, showing increased stress with higher strain rates due to dislocation interactions and decreased stress at higher temperatures due to dynamic recovery and recrystallization [54,55]. These findings provide a robust dataset for validating the proposed models and their accuracy in predicting material behavior under various forming conditions [56,57,58,59].

## 3. Physically Based Models

### 3.1. CP Modeling

As presented in Equation (1), F which describes the deformation gradient is decomposed to $F^{e}$ and $F^{p}$ [60,61]. The following conditions are necessary due to the incompressibility during plastic deformation, described as $J^{p}=det\;F^{p}=1$. Since $J^{e}=det\;F^{e}>0$, ***F^e^***, and ***F^p^*** are invertible.

(1) $$F=F^{e}F^{p}$$

Similarly, as explained in Equation (1), *L* describing the velocity gradient tensor was decomposed to $L^{e}$ and $L^{p}$, where $L^{p}=F^{e}{\tilde{L}}^{p}F^{e-1}$; thus, *L* is written as described in Equation (2):

(2) $$L=L^{e}+L^{p}={\dot{F}}^{e}F^{e-1}+F^{e}{\tilde{L}}^{p}F^{e-1}$$

The plastic flow can be expressed as outlined below:

(3) $${\tilde{L}}^{p}={\dot{F}}^{p}F^{p-1}=\sum_{\alpha -1}^{n}{{\dot{\gamma }}^{a}}^{S^{a}}$$

Elastic deformation is responsible for carrying the distortion of these slip systems. Thus, $m^{\alpha }$, and $n^{\alpha }$ in the present configuration is determined to their forms in $\tilde{\beta }$ by $F^{e}m_{0}^{\alpha }$ and $n_{0}^{\alpha }F^{e-1}$, respectively. Given that elastic deformation is minimal in comparison to plastic deformations, the following can be written:

(4) $$S^{e}=C^{e}:E^{e}=J^{e}F^{e-1}\sigma F^{e-T}$$

(5) $$E^{e}=(C^{e}-I)/2,\;C^{e}=F^{eT}F^{e}$$

$C^{e}$ is determined via the temperature-dependent stiffness parameters, written in Equation (6) as follows:

(6) $$C_{ij}=C_{ij}^{o}-\frac{S_{ij}}{\exp\left(\frac{t_{ij}}{\theta }\right)-1}$$

The parameters of temperature-dependent stiffness and elastic compliance are written in Table 1.

Thus, the following can be obtained:

(7) $${\dot{\gamma }}^{\alpha }=b\rho^{\alpha }{\bar{v}}^{\alpha }$$

Under quasistatic conditions, $\bar{v}$ is described below:

(8) $$\bar{v}≅l_{f}/t_{w}$$

where

(9) $${t_{w}}^{-1}=v_{D}\;b\;l_{f}\left[exp\left(-\frac{∆G^{*}}{k_{B}\theta }\right)\right]$$

Thus, the evolutions of the rate of crystallographic shearing are described as follows:

(10) $${\dot{\gamma }}^{a}={\dot{\gamma }}_{0}\;exp\left[\frac{F_{0}}{k_{B}\theta }{\left\{1-{\langle \frac{\left|\tau^{\alpha }\right|-S_{a}^{\alpha }}{{\hat{\tau }}^{\alpha }}\rangle }^{p}\right\}}^{q}\right]$$

Total dislocations’ densities are presented as the competition of the storage and recovery mechanisms as $\dot{\rho }={\dot{\rho }}^{\left(+\right)}+{\dot{\rho }}^{\left(-\right)}$. Assuming that dislocation nucleation is the dominant source, the rate of athermal storage is described using Equation (11):

(11) $${\dot{\rho }}^{\alpha }=\frac{c{\dot{\gamma }}^{\alpha }}{bL^{\alpha }}=2y_{c}\frac{\rho^{\alpha }{\dot{\gamma }}^{\alpha }}{b}$$

$\rho^{\alpha }$ is decomposed to $\rho_{e}^{\alpha }$ and $\rho_{s}^{\alpha }$. Thus, ${\dot{\rho }}_{i}^{\alpha }$ is described using Equation (12) as presented below:

(12) $${\dot{\rho }}_{i}^{\alpha }=\frac{C_{i}}{b}\left[\frac{K_{i}}{L_{i}}-2d_{i}\rho_{i}^{\alpha }\right]\left|{\dot{\gamma }}^{\alpha }\right|$$

$y_{s}$ is defined by Equation (13), which was proposed by Nix et al. [61]:

(13) $$y_{s}={\hat{y}}_{s}exp(-\frac{b}{a\theta })$$

Based on the Taylor equation [63,64,65,66], $S_{T}^{\alpha }$ is determined using Equation (14).

(14) $$S_{a}^{\alpha }=\lambda \mu b\sqrt{\sum_{\beta =1}^{N_{s}}h^{\alpha \beta }\rho^{\beta }}$$

For simplicity, $h^{\alpha \beta }$ is described using Equation (15) as follows:

(15) $$h^{\alpha \beta }=\omega_{1}+({1-\omega }_{2})\delta^{\alpha \beta }$$

**Figure 1:**
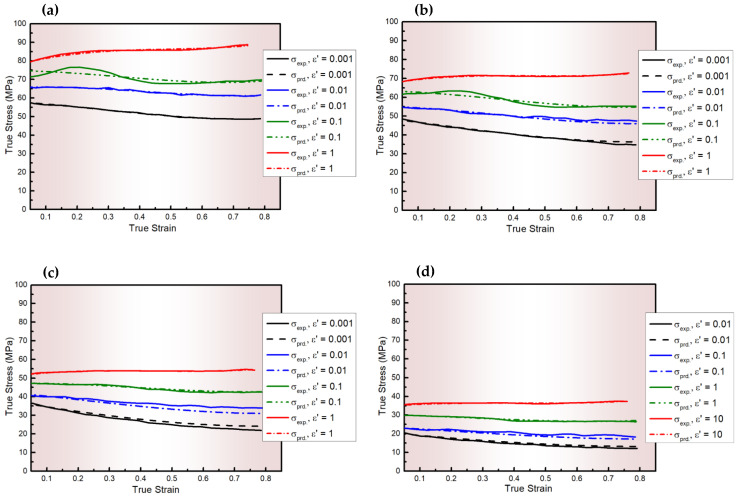
The experimental stresses of AA6082 and those predicted by CP modeling at $\dot{\varepsilon }$ values of 0.001–1 s^−1^ and T values of (**a**) 400, (**b**) 450, (**c**) 500, and (**d**) 550 °C.

To assess the predictability of the proposed CP model, the predicted and experimental results were compared as shown in Figure 1. The results indicate a high degree of agreement between the predicted and experimental stresses across all tested conditions. Further validation was conducted by calculating key statistical parameters including R, AARE, and RMSE [67,68] to evaluate the model’s reliability and quantify its predictive accuracy. These parameters were computed using Equations (16)–(18), with their respective values in Table 2. R indicates the strength of the linear relationship between observed and predicted values but it can be biased at extreme values; RMSE measures deviations from the mean, potentially influenced by outliers; in contrast, AARE provides a more reliable assessment by evaluating the average error magnitude relative to the actual values.

(16) $$R=\frac{\sum_{i=1}^{i=N}\left(\sigma_{E.}^{i}-{\bar{\sigma }}_{E})(\sigma_{P}^{i}-{\bar{\sigma }}_{P}\right)}{\sqrt{\sum_{i=1}^{i=N}{\left(\sigma_{E}^{i}-{\bar{\sigma }}_{E}\right)}^{2}\sum_{i=1}^{i=N}{\left(\sigma_{P}^{i}-{\bar{\sigma }}_{P}\right)}^{2}}}$$

(17) $$AARE\;(\%)=\frac{1}{N}\;\sum_{i=1}^{i=N}\left|\frac{\sigma_{E\;}^{i}-\sigma_{P}^{i}}{\sigma_{E.\;}^{i}}\right|\times 100$$

(18) $$RMSE=\sqrt{\frac{1}{N}\sum_{i=1}^{i=N}{\left(\sigma_{E}^{i}-\sigma_{P}^{i}\right)}^{2}}$$

### 3.2. Modified ZA (MZA) Constitutive Model

The ZA model [69] is a well-known physical model developed based on the principles of dislocation mechanisms. In this model, flow stresses are divided into athermal and thermal parts, as presented in Equation (19).

(19) $$\sigma =\sigma_{th}+\sigma_{a}$$

The ZA model offers a comparatively simple expression compared to other dislocation-based models. A key feature of the ZA model is that its formulation varies depending on the material’s structure (i.e., F.C.C. or B.C.C.), capturing the unique $\dot{\varepsilon }$ controlling mechanisms specific to each of these structures. The equations for the thermal activation flow stress are provided below.

(20) $$\sigma_{th}=C_{1}exp(-C_{3}T+C_{4}T\;ln\dot{\varepsilon })\;\;\;\;\;\;\;\;\;\;\;\;\;\;\;\;\;\;\;\;\;\;\;\;\;\;\;\;\mathrm{For}\;B.C.C.\mathrm{metals}$$

(21) $$\sigma_{th}=C_{2}\varepsilon^{1/2}(-C_{3}T+C_{4}T\;ln\dot{\varepsilon })\;\;\;\;\;\;\;\;\;\;\;\;\;\;\;\;\;\;\;\;\;\;\;\;\;\;\;\;\mathrm{For}\;F.C.C.\mathrm{metals}$$

By combining $\sigma_{th}\;$with the effect of yield stresses on grains sizes into a single component ($C_{0}$), Equations (20) and (21) are expressed as follows:

(22) $$\sigma ={C_{0}+C}_{1}exp(-C_{3}T+C_{4}T\;ln\dot{\varepsilon })+C_{5}\varepsilon^{n}\;\;\;\;\;\;\mathrm{For}B.C.C.\mathrm{metals}$$

(23) $$\sigma ={C_{0}+C}_{2}\varepsilon^{1/2}(-C_{3}T+C_{4}T\;ln\dot{\varepsilon })\;\;\;\;\;\;\;\;\;\;\;\;\;\;\;\;\;\;\;\;\;\;\mathrm{For}F.C.C.\mathrm{metals}$$

Although the ZA model incorporates dislocation mechanisms, it does not consider the impact of forming conditions, which diverge from those in practical forming processes, thereby reducing its predictive accuracy. To overcome these shortcomings, Samantaray et al. [69] introduced the MZA model expressed in Equation (24). The MZA model links softening with $\dot{\varepsilon }$ and ε to more accurately predict the flow behaviors of metals under hot working conditions. The MZA model thoroughly accounts for the effects of strain hardening, strain rate hardening, and thermal softening on the flow behaviors of metals under hot forming conditions, as introduced in Equation (24).

(24) $$\sigma =\left(C_{1}+C_{2}\varepsilon^{n}\right)\exp\left[-\left(C_{3}+C_{4}\varepsilon \right)T^{*}+\left(C_{5}+C_{6}T^{*}\right)\ln\varepsilon^{\cdot *}\right]$$

To determine the constants for the MZA model, the corresponding equations are rearranged to isolate the constants on the right-hand side. Although the relationships between the predictors ε, $\varepsilon^{\cdot }$, and $T^{*}$ and the response (the equation’s output on the left-hand side) are nonlinear, the equations are linear with respect to the constants (coefficients). Therefore, a linear regression model using the least-squares method is employed to compute the constants for the three models in MATLAB.

To obtain the model’s constants of the current study, $T_{r}$ and ${\dot{\varepsilon }}_{r}$ are set as 400 °C and 0.001 s^−1^. Consequently, Equation (24) simplifies to the following:

(25) $${\sigma =C}_{1}+C_{2}\varepsilon^{n}$$

where $C_{1}$ is measured as 58 MPa. By regression, the n and $C_{2}$ were determined to be 0.847 and −12.738 MPa, respectively.

After modifications and at $\dot{\varepsilon }$ = 0.001 s^−1^, Equation (24) is written as outlined below:

(26) $$\ln\left[\frac{\sigma }{C_{1}+C_{2}\varepsilon^{n}}\right]=\left[-\left(C_{3}+C_{4}\varepsilon \right)\right]T^{*}$$

Through regression analysis, $C_{3}$ and $C_{4}$ were determined to be −0.0049 and −0.0041, respectively. After rearrangements, Equation (24) is reformulated as presented in Equation (27).

(27) $$\ln\left[\frac{\sigma }{C_{1}+C_{2}\varepsilon^{n}}\right]+\left(C_{3}+C_{4}\varepsilon \right)T^{*}=\left(C_{5}+C_{6}T^{*}\right)\ln\varepsilon^{\cdot *}$$

The other values of T and $\dot{\varepsilon }$ were then utilized to calculate $C_{5}$ and $C_{6}$ through regression as 0.0923 and −0.00005. All of the MZA model’s constants of the AA6082 Al-Mg-Si alloy are presented in Table 3.

Therefore, the MZA model of the AA6082 alloy is written as follows:

(28) $$\sigma =\left(58-12.738\varepsilon^{0.847}\right)\exp\left[-\left(0.0049-0.0041\varepsilon \right)T^{*}+\left(0.0923-0.00005T^{*}\right)\ln\varepsilon^{\cdot *}\right]$$

**Figure 2:**
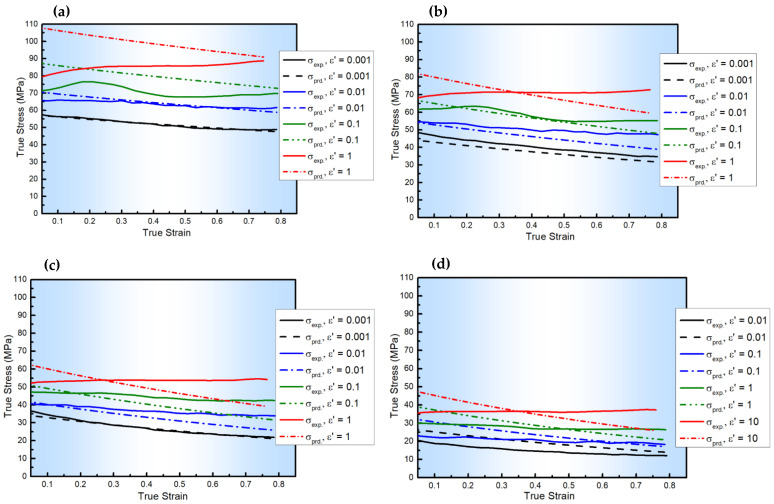
The experimental stresses of AA6082 and those predicted by the MZA model at $\dot{\varepsilon }$ values of 0.001–1 s^−1^ and T values of (**a**) 400, (**b**) 450, (**c**) 500, and (**d**) 550 °C.

Figure 2 shows the comparison between the experimental flow behaviors of AA6082 Al-Mg-Si and those predicted by the MZA model, with R = 0.951, AARE = 11.67%, and RMSE = 7.23 MPa, as listed in Table 4. As depicted, the MZA model shows a good level of accuracy for determining the flow stresses under hot conditions across all given $\dot{\varepsilon }$ values, notably at or near $T_{r}$ and ${\dot{\varepsilon }}_{r}$. This level of accuracy is due to the model’s integration of isotropic hardening, strain rate hardening, and thermal softening, along with the combined influence of T, $\dot{\varepsilon }$, and ε on the flow stresses.

## 4. Phenomenological Models

### 4.1. Shokry’s MJC-1 (S1-MJC)

The MJC model developed by Shokry [70] is modified in this study to improve its accuracy in predicting the deformation behavior of AA6082 at several values of T and $\dot{\varepsilon }$ by incorporating a linear relationship that directly links ε with T and $\dot{\varepsilon }$. The S1-MJC model is formulated below:

(29) $$\sigma =\left(A+{B_{1}\varepsilon +B_{2}\varepsilon }^{2}+{B_{3}\varepsilon }^{3}\right)\left(1+\left(C_{1}+C_{2}\varepsilon \right)\ln\varepsilon^{\cdot *}\right)\left(1-{T^{*}}^{\left(m+m_{2}\varepsilon \right)}\right)$$

For obtaining the S1-MJC model’s constants, $T_{r}$ and ${\dot{\varepsilon }}_{r}$ are set as 400 °C and 0.001 s^−1^. Therefore, Equation (29) simplifies to the following:

(30) $$\sigma =A+{B_{1}\varepsilon +B_{2}\varepsilon }^{2}+{B_{3}\varepsilon }^{3}$$

Through regression analysis, A was determined to be 58.071 MPa, $B_{1}$ was −11.065 MPa, $B_{2}$ was −22.881 MPa, and $B_{3}$ was 27.824 MPa.

After modifications and at T = 400 °C, Equation (29) is described below:

(31) $$\frac{\sigma }{A+{B_{1}\varepsilon +B_{2}\varepsilon }^{2}+{B_{3}\varepsilon }^{3}}-1=\left(C_{1}+C_{2}\varepsilon \right)\ln\varepsilon^{\cdot *}$$

Using regression, $C_{1}$ was determined to be 0.0616 and $C_{2}$ was 0.0641. After applying the natural logarithm and simplifying, Equation (29) can be expressed in various $\dot{\varepsilon }$ values as follows:

(32) $$\ln\left[1-\frac{\sigma }{\left(A+{B_{1}\varepsilon +B_{2}\varepsilon }^{2}+{B_{3}\varepsilon }^{3}\right)\left(1+\left(C_{1}+C_{2}\varepsilon \right)\ln\varepsilon^{\cdot *}\right)}\right]=\left(m_{1}+m_{2}\varepsilon \right){\;T}^{*}$$

Using regression, $m_{1}$ was determined to be 1.4884 and $m_{2}$ was determined to be −0.3467. The S1-MJC model constants of the AA6082 alloy are written in Table 5.

Therefore, the S1-MJC model of AA6082 alloy is presented below:

(33) $$\sigma =\left(58.071-11.065{\varepsilon -22.881\varepsilon }^{2}+{27.824\varepsilon }^{3}\right)\left(1+\left(0.0616+0.0641\varepsilon \right)\ln\varepsilon^{\cdot *}\right)\left(1-{T^{*}}^{\left(1.4884-0.3467\varepsilon \right)}\right)\;\;$$

**Figure 3:**
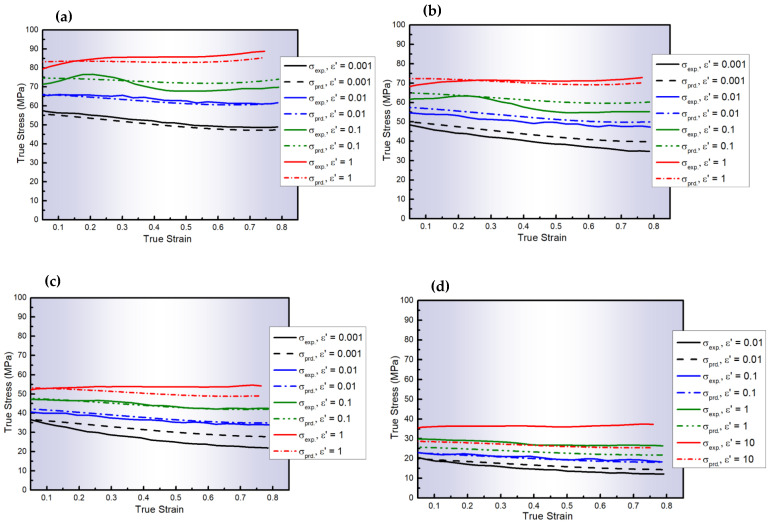
The experimental stresses of AA6082 and those predicted by the S1-MJC model at $\dot{\varepsilon }$ values of 0.001–1 s^−1^ and T values of (**a**) 400, (**b**) 450, (**c**) 500, and (**d**) 550 °C.

Figure 3 compares the experimental flow stresses of AA6082 and those determined via the S1-MJC model, with R = 0.983, AARE = 7.09%, and RMSE = 3.56 MPa, as listed in Table 6. As depicted, the results predicted by the S1-MJC model align closely with the experimental data, demonstrating a better fit than the MZA model; however, the S1-MJC model still does not outperform the accuracy achieved with CP modeling. This improved accuracy is because the S1-MJC model integrated both $\dot{\varepsilon }$ and the softening effects and strain hardening in the model. It is well-established that dislocations are influenced by $\dot{\varepsilon }$ and softening.

### 4.2. Shokry’s MJC-2 (S2-MJC)

The MJC model developed by Shokry et al. [71] is implemented in this study for determining the hot-flow behavior of AA6082 Al-Mg-Si over a wide range of T and $\dot{\varepsilon }$. Their model is represented below:

(34) $$\sigma =\left(\sum_{i=0}^{3}A_{i}\varepsilon^{i}\right)\left(1+\left(\sum_{i=0}^{2}\sum_{j=0}^{2}C_{ij\;}\varepsilon^{i}\varepsilon^{\cdot j}\right)\ln\varepsilon^{\cdot *}\right)\exp\left[\left(\sum_{i=0}^{2}\sum_{j=0}^{2}\sum_{k=0}^{2}{m_{ijk}\;\varepsilon }^{i}\varepsilon^{\cdot j}T^{*k}\right)T^{*}\right]$$

In this investigation, to determine the S2-MJC model’s constants, ${\dot{\varepsilon }}_{r}$ was adjusted to 0.001 s^−1,^ and $T_{r}$ was adjusted to 400 °C. Consequently, Equation (34) is simplified to the following:

(35) $$\sigma =\sum_{i=0}^{3}A_{i}\varepsilon^{i}$$

Expanding Equation (35) results in four parts including ε, each associated with four constants. Through regression, these constants were 58.071 MPa, −11.065 MPa, −22.881 MPa, and 27.824 MPa. Thus, at 400 °C, and after performing some adjustments, Equation (34) is presented below:

(36) $$\left(\frac{\sigma }{\sum_{i=0}^{3}A_{i}\varepsilon^{i}}-1\right)/\ln\varepsilon^{\cdot *}=\sum_{i=0}^{2}\sum_{j=0}^{2}C_{ij\;}\varepsilon^{i}\varepsilon^{\cdot j}$$

Expanding Equation (36) results in nine parts, including $\dot{\varepsilon }$ and ε, each linked to a corresponding constant. Through regression, these constants were 0.0550, 0.0828, −0.0888, 0.1637, −1.2841, 1.2652, −0.1126, 1.0707, and −1.0288. After applying the natural logarithm and making adjustments, Equation (34) can be expressed in various $\dot{\varepsilon }$ values as follows:

(37) $$\frac{\ln\left[\frac{\sigma }{\left(\sum_{i=0}^{3}A_{i}\varepsilon^{i}\right)\left(1+\left(\sum_{i=0}^{2}\sum_{j=0}^{2}C_{ij\;}\varepsilon^{i}\varepsilon^{\cdot j}\right)\ln\varepsilon^{\cdot *}\right)}\right]}{T^{*}}=\sum_{i=0}^{2}\sum_{j=0}^{2}\sum_{k=0}^{2}{m_{ijk}\;\varepsilon }^{i}\varepsilon^{\cdot j}T^{*k}$$

Expanding the right side of Equation (37) results in 27 parts, including T, $\dot{\varepsilon }$, and ε. Through regression, these constants were obtained to be −0.6668, 2.7593, −2.4307, −0.2755, −5.5232, 5.0762, −0.1888, 2.1973, −1.5661, −0.0198, −10.3673, 9.3805, −2.597, 56.627, −53.125, 2.2348, −35.0907, 32.2649, −1.6642, 16.4578, −14.7332, 3.5334, −73.087, 68.9637, −2.7157, 52.349, and −49.0335, as listed in Table 7.

To determine the constants for the SI-MJC and S2-MJC models, the corresponding equations are rearranged to isolate the constants on the right-hand side. Although the relationships between the predictors ε, $\varepsilon^{\cdot }$, and $T^{*}$ and the response (the equation’s output on the left-hand side) are nonlinear, the equations are linear with respect to the constants (coefficients). Therefore, a linear regression model using the least-squares method is employed to compute the constants for the three models in MATLAB.

**Figure 4:**
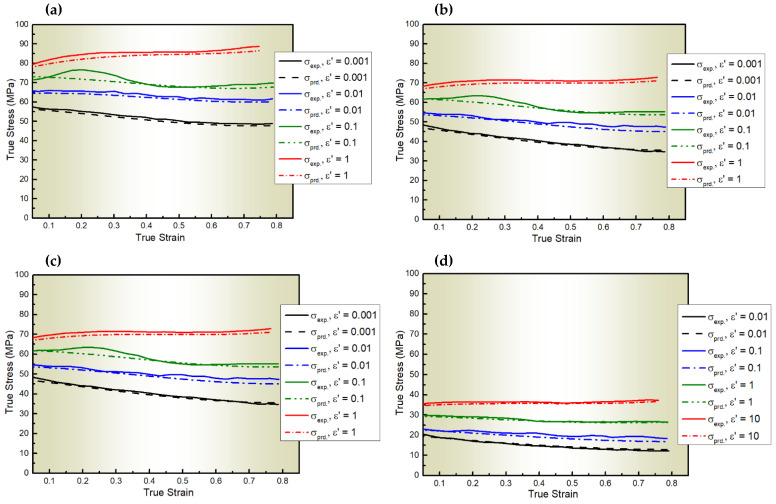
The experimental stresses of AA6082 and those predicted by the S2-MJC model at $\dot{\varepsilon }$ values of 0.001–1 s^−1^ and T values of (**a**) 400, (**b**) 450, (**c**) 500, and (**d**) 550 °C.

Figure 4 shows comparisons between the experimental stresses of AA6082 and those determined via the S2-MJC model, with R = 0.99, AARE = 1.87%, and RMSE = 0.95 MPa. As depicted in Figure 4 and Table 8, the result predicted by the S2-MJC model closely aligns with the experimentation, showing a better fit than the previous models and close to the accuracy achieved with CP modeling. This improved accuracy is because the S2-MJC model integrated both $\dot{\varepsilon }$ and ε on the one hand, and T, $\dot{\varepsilon }$, and ε on the other hand, facilitated by an extensive set of constants that link the softening and dynamic recovery components.

**Figure 5:**
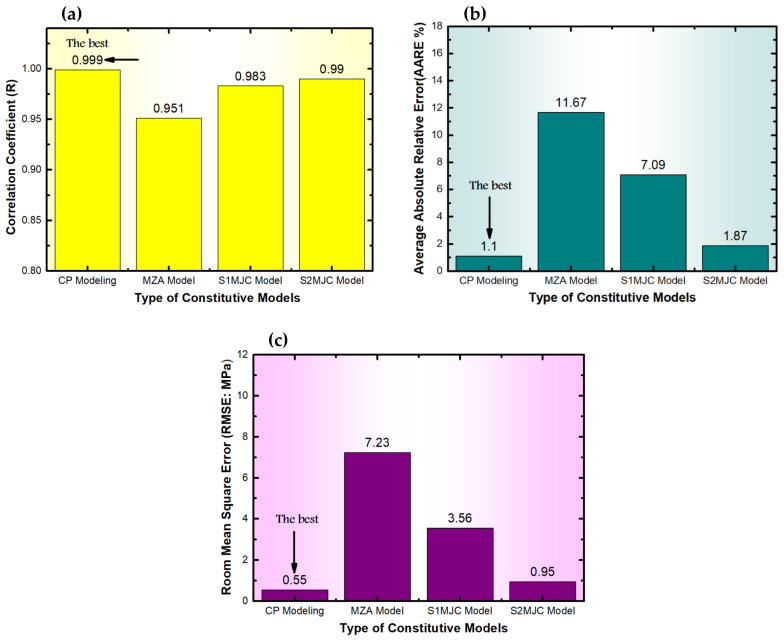
Comparisons between the proposed models’ (**a**) R, (**b**) AARE, and (**c**) RMSE for verifying their predictability.

The *R* values for the developed models are presented in Figure 5a. The CP and S2-MJC models achieve the highest *R* values close to 1, with values of 0.999 and 0.99, respectively. In contrast, *R* for the MZA is 0.951 and for S1-MJC is 0.983. Similarly, Figure 5b,c display these models’ *AARE* and *RMSE* values. The CP and S2-MJC models exhibit the best performance with the lowest values of *AARE*, which are 1.1% and 1.87%, respectively. The values of *RMSE* are 0.55 MPa and 0.95 MPa, respectively. Moreover, the MZA and S1-MJC models yield higher *AARE* values of 11.67% and 7.09% and *RMSE* values of 7.23 MPa and 3.56 MPa, respectively. The obtained values of *R*, *AARE*, and *RMSE* indicate that the MZA and S1-MJC models can be used to predict the flow behavior of the studied alloy but with limited accuracy. The MZA model accounts for the coupling effects between ε and T as well as between $\dot{\varepsilon }$ and T. In contrast, the S1-MJC model considers the coupling effects between ε and T as well as between ε and $\dot{\varepsilon }$. Moreover, the CP and S2-MJC models incorporate the coupling effects between T, $\dot{\varepsilon }$, and ε. Given the nonlinear nature of the flow behavior of the studied alloy, models that account for the more comprehensive coupling between T, $\dot{\varepsilon }$, and ε are expected to provide more accurate predictions.

## 5. Conclusions

The hot-flow behaviors of Al-Mg-Si alloy are complex due to their reliance on ***ε***, $\dot{\varepsilon }$, and T; thus, determining its deformation behavior across a broad range of forming conditions is crucial. Consequently, CP modeling and the MZA, S1-MJC, and S2-MJC models were developed in this investigation to predict the hot deformation behavior of Al-Mg-Si alloy precisely. The accuracy of these models was evaluated via comparisons of the predicted and experimental results at $\dot{\varepsilon }$ values of 0.001–10 s^−1^ and T values of 400–550 °C. Additional validations were performed by calculating statistical parameters to assess the accuracy of the developed models. Through comparing the experimental and determined stresses and confirming these findings through the statistical values, it is concluded that the flow stresses predicted by the CP and S2-MJC models closely align with the experimentation stresses. This contrasts with the results from the MZA and S1-MJC models. These findings are attributed to the ability of CP modeling to couple the microstructure state of the Al-Mg-Si alloy with its mechanical behavior. In addition, the ability of the S2-MJC model to integrate both $\dot{\varepsilon }$ and ε on the one hand, and T, $\dot{\varepsilon }$, and ε on the other hand, is facilitated by extensive constants that link the softening and dynamic recovery components.

## Figures and Tables

**Table 1:** The temperature-dependent stiffness and elastic compliance parameters.

$C_{11}^{0}\;(GPa)$	$C_{12}^{0}\;(GPa)$	$S_{44}=GPa$	$S_{12}\;(GPa)$	$S_{11}\;(GPa)$	$t_{11}$	$t_{12}$	$t_{44}$
114.2	61.9	2.56	2.0	10.1	258.4	293.6	168

**Table 2:** The values of statistical parameters of the proposed CP modeling of the AA6082 Al-Mg-Si alloy.

R	AARE (%)	RMSE (MPa)
0.999	1.1%	0.55

**Table 3:** MZA model constants of the AA6082 Al‐Mg‐Si alloy.

$C_{1}\;[MPa]$	$C_{2}\;[MPa]$	n	$C_{3}$	$C_{4}$	$C_{5}$	$C_{6}$
58	−12.738	0.847	−0.0049	−0.0041	0.0923	−0.00005

**Table 4:** The values of statistical parameters of the MZA model of the AA6082 Al-Mg-Si alloy.

R	AARE (%)	RMSE (MPa)
0.951	11.67%	7.23

**Table 5:** S1-MJC model constants of AA6082 alloy.

A [MPa]	$B_{1}\;[MPa]$	$B_{2}\;[MPa]$	$B_{3}\;[MPa]$	$C_{1}$	$C_{2}$	$m_{1}$	$m_{2}$
58.071	−11.065	−22.881	27.824	0.0616	0.0641	1.4884	−0.3467

**Table 6:** The values of statistical parameters of the modified S1-MJC model of the AA6082 alloy.

R	AARE (%)	RMSE (MPa)
0.983	7.09%	3.56

**Table 7:** S2-MJC model constants of the AA6082 Al-Mg-Si alloy.

$A_{0}$ [MPa]	$A_{1}$ [MPa]	$A_{2}$ [MPa]	$A_{3}$ [MPa]	$C_{00}$	$C_{01}$	$C_{02}$	$C_{10}$
58.071	−11.065	−22.881	27.824	0.0550	0.0828	−0.0888	0.1637
$C_{11}$	$C_{12}$	$C_{20}$	$C_{21}$	$C_{22}$	$m_{000}$	$m_{001}$	$m_{002}$
−1.2841	1.2652	−0.1126	1.0707	−1.0288	−0.6668	2.7593	−2.4307
*m*010	*m*011	*m*012	*m*020	*m*021	*m*022	*m*100	*m*101
−0.2755	−5.5232	5.0762	−0.1888	−2.1973	−1.5661	−0.0198	10.3673
$m_{102}$	$m_{110}$	$m_{111}$	$m_{112}$	$m_{120}$	$m_{121}$	$m_{122}$	$m_{200}$
−9.3805	2.597	−56.627	53.125	−2.2348	35.0907	32.2649	−1.6642
$m_{201}$	$m_{202}$	$m_{210}$	$m_{211}$	$m_{212}$	$m_{220}$	$m_{221}$	$m_{222}$
16.4578	14.7332	−3.5334	73.087	68.9637	−2.7157	52.349	−49.0335

**Table 8:** The values of statistical parameters of the proposed S2-MJC models of the AA6082 alloy.

R	AARE (%)	RMSE (*MPa*)
0.99	1.87%	0.95

## Data Availability

The original contributions presented in the study are included in the article, further inquiries can be directed to the corresponding author.

## Nomenclature

Symbol	Abbreviations
T	Temperature
$\dot{\varepsilon }$	Strain rate
F	Deformation gradient
$F^{e}$	Elastic component of deformation gradient
$F^{p}$	Plastic component of deformation gradient
L	Velocity gradient tensor
$L^{e}$	Elastic component of velocity gradient tensor
$L^{p}$	Plastic component of velocity gradient tensor
${\tilde{L}}^{p}$	$\mathrm{pull}-\mathrm{back}\;\mathrm{of}\;\mathrm{the}\;\mathrm{plastic}\;\mathrm{velocity}\;\mathrm{gradient}\;\mathrm{to}\;\tilde{\beta }$
$\tilde{\beta }$	locally stress-free intermediate configuration
${\dot{\gamma }}^{a}$	shearing rate on the *α*-slip system
n	potential slip system’s number
$S^{a}$	Schmid tensor ${(=m}_{0}^{a}\;⮾\;n_{0}^{a})$, $m_{0}^{\alpha }$ and $n_{0}^{\alpha }$ are orthogonal to each other
$m_{0}^{a}$	Slip plane direction
$n_{0}^{a}$	Slip plane normal
$m^{\alpha }$	Slipping direction
$n^{\alpha }$	Equivalent slip plane normal
$S^{e}$	The second Paola-Kirchhoff stress tensor
σ	Cauchy stress tensor
E	The elastic Green-Lagrange strain tensor
$C^{e}$	The elastic right Cauchy-Green deformation tensor
I	Identity tensor
θ	The absolute temperature (K)
$\tau^{\alpha }$	The resolved shear stress tensor
b	The magnitude of the Burgers vector
$\rho^{\alpha }$	The mobile dislocations density on the α slip system.
${\bar{v}}^{\alpha }$	The average velocity of mobile dislocations on the α slip system.
$\bar{v}$	The average dislocation velocity
$l_{f}$	The average distance between each obstacle on the slip plane.
$t_{w}$	The waiting time for a dislocation to surpass the local obstacles.
$v_{D}$	The Debye frequency
$k_{B}$	The Boltzmann constant
$∆G^{*}$	The difference in the activation-free enthalpy when the dislocation segment moves from the stable configuration to the unstable configuration
${\dot{\gamma }}_{0}$	The reference slip rate
c	The fraction of the shearing rate by the total dislocation
L	The mean free path
$y_{c}$	The critical annihilation distance for canceling out the two dislocations with opposite polarities
$\rho^{\alpha }$	The total dislocation density
$\rho_{e}^{\alpha }$	Edge dislocation component
$\rho_{s}^{\alpha }$	Screw dislocation component
${\dot{\rho }}_{i}^{\alpha }$	The dislocation density evaluation, where: i=e,s
${\hat{y}}_{s}$	The reference critical annihilation distance
a	Constant = 0.001
b	Constant = 8.617E-5
$S_{T}^{\alpha }$	The total athermal slip resistance parameters
b	The Burgers vector’s magnitude
μ	The shear modulus,
λ	For identifying the deviations from the regular spatial arrangements of dislocation density
$h^{\alpha \beta }$	The matrix of dislocation interaction
$\delta^{\alpha \beta }$	The Kronecker delta
$\omega_{1}$, $\omega_{2}$	The interaction coefficients
R	correlation coefficient
AARE	average absolute relative error
RMSE	root mean square error
N	The total number of points included in the analysis
$\sigma_{E}^{i}$	The experimental stress values
$\sigma_{P}^{i}$	The predicted stress values
${\bar{\sigma }}_{E}$	The mean values of experimental stress
${\bar{\sigma }}_{P}$	The mean values of predicted stress
$\sigma_{a}$	The athermal activation flow stress
$\sigma_{th}$	The thermal activation flow stress.
BCC	Body-centered cubic
FCC	face-centered cubic
$C_{0},C_{1}$, $C_{2}$, $C_{3}$, $C_{4}{,C}_{5}$, n	material constants
T	The testing temperature
σ	Von-Mises flow stress
ε	equivalent plastic strain
${\dot{\varepsilon }}_{r}$	reference strain rates
$\varepsilon^{\cdot *}$	the ratio between the testing $\dot{\varepsilon \;}$ and ${\dot{\varepsilon }}_{r}$ ($\varepsilon^{\cdot *}=\frac{\dot{\varepsilon \;}}{{\dot{\varepsilon }}_{r}}$)
$T_{r}$	Reference temperatures
$T^{*}$	The difference between testing T and $T_{r}$ ($T^{*}=$$T-T_{r})$
$m_{2}$, $m_{1},C_{2},C_{1},B_{3},B_{2},B_{1},$A	material constants
${T^{*}}_{S1}$	${T^{*}}_{S1}=\frac{T-T_{r}}{T_{m}-T_{r}}$
$T_{m}$	The melting temperature of AA6082 Al-Mg-Si
$A_{i}$	constants correlate with the $\varepsilon^{i}$
$\varepsilon^{i}$	representing the strain-hardening component
$C_{ij}$	constants associate with the $\varepsilon^{\cdot *}$
$m_{ijk}$	constants related to the softening parameter $T^{*}$
